# Drowned

**Published:** 1884-09

**Authors:** 


### Drowned.

Allison Wright, oldest son of Dr. A. W. and Bessie M. Harlan, aged nine years, was drowned at Crystal Lake, near Chicago, on Tuesday, August 19th. This sad announcement, taken from a Chicago paper of the 20th, will bring sadness to the hearts of Dr. Harlan's many warm friends, and he will certainly have the symyathy of all his professional brethren in this his great bereavement.

